# Controlled Irrigation Improves Nitrogen Partitioning and Agronomic Nitrogen Use Efficiency in Rice Under Moderate Nitrogen Inputs

**DOI:** 10.3390/plants15050739

**Published:** 2026-02-28

**Authors:** Haijun Liu, Tangzhe Nie, Peng Chen, Lili Jiang, Tianyi Wang, Anis Ur Rehman Khalil, Susumu Miyazu

**Affiliations:** 1 School of Water Conservancy and Electric Power, Heilongjiang University, Harbin 150080, China; 2232052@s.hlju.edu.cn (H.L.);; 2 College of Agricultural Science and Engineering, Hohai University, Nanjing 211100, China; 3 State Key Laboratory of Water Disaster Prevention, Hohai University, Nanjing 211100, China; 4 College of Hydrology and Water Resources, Hohai University, Nanjing 211100, China; 5 Faculty of Agriculture, Niigata University, Niigata 9502181, Japan

**Keywords:** agronomic nitrogen use efficiency, nitrogen partitioning, *Oryza sativa* L., water–nitrogen interaction

## Abstract

Nitrogen (N) application rates and irrigation regimes are key factors determining rice yield and N use efficiency. To evaluate the effects of different irrigation regimes and N application rates on rice yield and N uptake, a four-year field experiment was conducted from 2021 to 2024 at the Qing’an National Irrigation Experimental Station in Heilongjiang Province. The experiment included two irrigation regimes (C: Controlled irrigation and F: Flooded irrigation) combined with four N application rates (N0: 0 kg N·ha^−1^, N1: 82.5 kg N·ha^−1^, N2: 110 kg N·ha^−1^, and N3: 137.5 kg N·ha^−1^). The results showed that, considering the same N application rate, C promoted dry matter accumulation by 3% to 9% and total N accumulation by 4.1% to 25.5% in the aboveground parts of rice compared to F. Under the same irrigation regime, total N accumulation in the aboveground parts of rice increased with N application rate and then plateaued. Regarding the distribution of N among organs, the proportion of panicle N relative to total N in the aboveground parts of rice followed an initial increase and subsequent decline as N input increased, resulting in the nitrogen harvest index (NHI) reaching its maximum under the moderate N treatment (N2). Overall, controlled irrigation significantly improved the NHI and AE, whereas the moderate N treatment (N2) further increased the NHI and promoted greater N allocation to panicles. Therefore, combining C with a moderate N application rate can enhance N use efficiency and markedly improve the internal N partitioning pattern.

## 1. Introduction

Rice (*Oryza sativa* L.) is one of the most important staple crops worldwide and serves as the primary food source for more than half of the global population [1]. Ensuring sustainable and stable rice production is, therefore, crucial to global food security [2]. In recent decades, population growth, industrial expansion, environmental pollution, and global climate change have intensified water scarcity for agricultural irrigation and increased N losses from croplands, thereby posing serious threats to global rice production [3,4]. Under conditions of limited irrigation resources and increasingly strict N control requirements, maintaining or enhancing rice yield has become a major challenge for sustainable rice production systems [5].

Continuous flooded irrigation is a traditional irrigation regime that provides a relatively stable soil environment for rice and typically ensures high yields. However, this practice requires substantial amounts of irrigation water and N fertilizer, often resulting in low resource use efficiency and increased environmental pressure [6,7,8]. In contrast, water-saving irrigation practices have demonstrated strong potential to reduce water input and N loss while sustaining rice productivity. Among these practices, controlled irrigation is commonly recognized as a representative water-saving irrigation method in paddy rice systems [9]. Compared with traditional flooded irrigation, water-saving irrigation can reduce N losses from paddy fields through runoff, leaching, and volatilization, while promoting N transformation processes such as mineralization and nitrification, thereby improving N availability in the rhizosphere. These processes provide a physiological basis for sustaining yields under reduced N inputs [10,11,12,13]. The N application rate is another crucial factor regulating rice yield and N use efficiency. Previous studies have shown that yield responses to increasing N rates often exhibit a plateau or diminishing marginal returns [7]. Excessive N fertilization can delay the senescence of vegetative organs, increase N retention in stems and leaves, and reduce the translocation of N and its assimilation into the grains, ultimately leading to significant reductions in grain N concentration and yield [14].

Rice yield depends not only on the total amount of N absorbed by the plant but also on the distribution of N among leaves, stems, and panicles [15]. The nitrogen harvest index (NHI) is widely used as an indicator to characterize the efficiency of N translocation from vegetative organs to the panicle [16]. N accumulation in the panicle and its remobilization from vegetative organs are directly associated with grain filling and protein formation in rice [17]. Controlled irrigation modifies soil moisture conditions and rhizosphere redox status, which can influence N mineralization, root N uptake, and N allocation during the reproductive growth stage [11]. These effects suggest that irrigation regimes influence not only total N accumulation but also N partitioning patterns among plant organs, thereby affecting the N harvest index [18]. In rice production, improving N use efficiency by optimizing plant N allocation and reducing the need for high N rates to sustain yield has become a central scientific challenge. However, existing studies have primarily focused on short-term responses or single management factors, and robust long-term field evidence remains limited regarding the quantitative relationships among irrigation regime, N application rate, nitrogen partitioning efficiency, and grain yield formation across multiple growing seasons. In particular, it remains unclear how irrigation-induced changes in N allocation among vegetative and reproductive organs translate into sustained yield performance and agronomic N use efficiency over time [19]. Furthermore, in paddy systems, irrigation methods and N inputs may induce gradual changes in soil physicochemical properties and root–soil interactions, which often exhibit time-lagged responses. Therefore, a long-term experimental approach provides a more robust and realistic assessment of the agronomic and environmental consequences of different water and N management strategies.

This study aims to clarify how irrigation regime and N application rate jointly shape N accumulation and allocation among rice organs. The objectives of this study were to (i) characterize the effects of irrigation regimes and N application rates on N uptake and allocation in rice, and (ii) quantify whether improved N allocation under controlled irrigation mitigates the dependence of rice yield on high N input while sustaining yield stability and nitrogen use efficiency. The findings are expected to enhance understanding of water–nitrogen interactions in rice systems and support the development of resource-efficient cultivation strategies.

## 2. Results

### 2.1. Rice Yield

**Figure 1:**
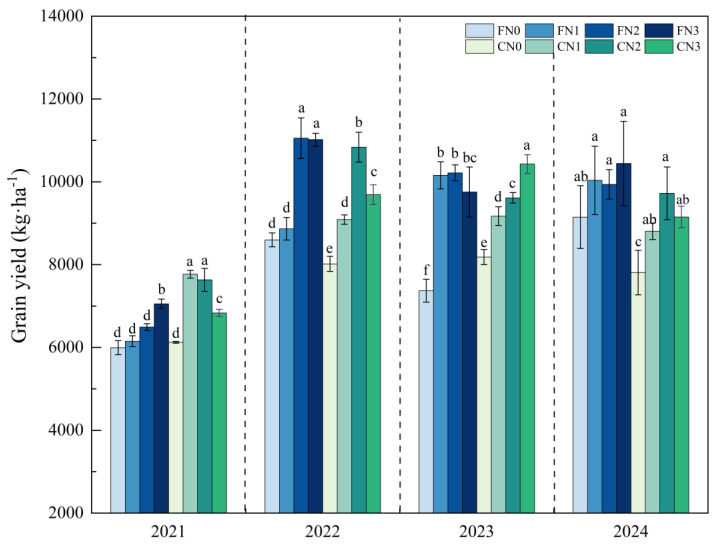
Rice yield under different treatments during 2021–2024. Error bars indicate the standard errors of the means (n = 3); Different lowercase letters indicate significant differences among treatments within the same year (*p* < 0.05). C: Controlled irrigation, F: flooded irrigation. Under each irrigation regime, four nitrogen application rates of 0 (N0), 82.5 (N1), 110 (N2), and 137.5 (N3) kg N·ha^−1^ were applied.

Rice yield under F was significantly higher than that under C by 2–12% (*p* < 0.05, Table 1). The yield response to nitrogen application rate differed between irrigation regimes. Under controlled irrigation, rice yield generally exhibited an increasing trend with increasing nitrogen application rate, followed by a tendency to decline at higher nitrogen application rates. Under F, rice yield increased with nitrogen application rate and then plateaued (Figure 1). The N2 treatment produced relatively high and stable yields, and further increases in nitrogen application did not result in additional yield gains.

### 2.2. Rice Aboveground Dry Matter Accumulation

Within each irrigation regime, aboveground dry matter accumulation of rice increased with increasing nitrogen application rate (N0–N2) but declined at the highest nitrogen application rate (N3) (Table 2). The N2 treatment achieved the highest rice aboveground dry matter accumulation, exceeding the other treatments by 3.2% to 20.9%. At the same nitrogen application rate, C resulted in significantly higher rice aboveground dry matter accumulation than F, with increases of 3% to 9% (Table 2 and Table 3).

Across treatments, dry matter accumulation allocation consistently followed the order panicle > stem > leaf. With increasing nitrogen application, dry matter accumulation in panicles and stems increased significantly, whereas changes in leaf dry matter were relatively small. Panicle dry matter increased with increasing nitrogen application rate up to a threshold level, beyond which further nitrogen input (N3) led to stagnation or a decline. At the same nitrogen application rate, panicle dry matter under C was significantly higher than that under F in most years, with increases ranging from 3.2% to 26.4%. Overall, the CN2 treatment exhibited higher dry matter accumulation with lower interannual variability and showed a relatively greater proportion of dry matter allocated to panicles.

### 2.3. Plant Aboveground N Accumulation

Plant aboveground N accumulation showed a similar pattern to dry matter accumulation (Table 4). Plant aboveground N accumulation increased from N0 to N2 and plateaued or varied at N3 depending on year and irrigation regime. At the same nitrogen application rate, total aboveground nitrogen accumulation under C was significantly higher than that under F in most years, with increases ranging from 4.1% to 25.5%. These results indicate that C effectively enhanced nitrogen uptake in rice plants (Table 5). However, as nitrogen input increased, the marginal increase in nitrogen accumulation declined at N3, suggesting a reduced plant response to excessive nitrogen application.

### 2.4. Nitrogen Partitioning Among Aboveground Organs

**Figure 2:**
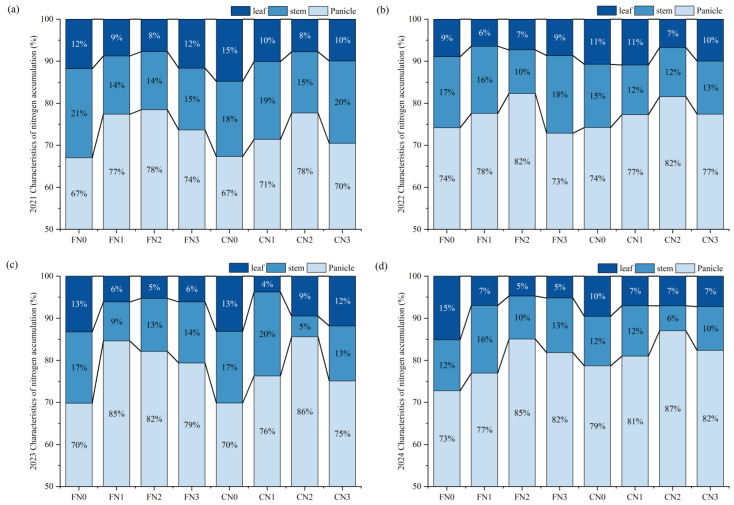
Distribution of nitrogen accumulation (%) among rice organs (leaf, stem, and panicle) under different treatments in (**a**) 2021, (**b**) 2022, (**c**) 2023, and (**d**) 2024. C: Controlled irrigation, F: flooded irrigation. Under each irrigation regime, four nitrogen application rates of 0 (N0), 82.5 (N1), 110 (N2), and 137.5 (N3) kg N·ha^−1^ were applied.

Across all treatments, the proportion of nitrogen accumulation among aboveground organs consistently followed the order of panicle > stem > leaf (Figure 2). The panicle served as the primary sink for nitrogen, accounting for 67% to 85% of the total aboveground nitrogen in rice plants. In contrast, stems and leaves contributed 14% to 21% and 4% to 15%, respectively.

With increasing nitrogen application rate, the proportion of nitrogen allocated to the panicle initially increased and then declined. Under the highest nitrogen application rate (N3), a greater proportion of nitrogen was retained in vegetative organs (stems and leaves). At the same nitrogen application rate, panicle nitrogen accumulation was generally higher under C than under F across different nitrogen fertilization treatments.

### 2.5. Agronomic Efficiency of Nitrogen Fertilizer

In this study, the AE generally increased with increasing nitrogen application up to N2, followed by a decline at N3; however, under flooded irrigation, some years showed a plateau or stagnation in AE at higher nitrogen rates (Table 6). At the same nitrogen application rate, the AE under C was generally higher than that under F, with the advantage most evident at the N2 treatment. Furthermore, compared with F, C tended to show less interannual fluctuation in AE, particularly under the N2 treatment, implying enhanced stability. These findings indicate that CN2 may contribute to improved and more stable agronomic nitrogen use efficiency.

### 2.6. Harvest Index and Nitrogen Harvest Index

**Figure 3:**
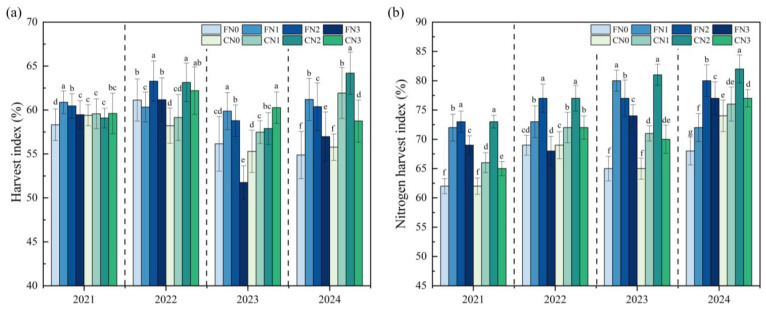
Harvest index (HI) (**a**) and nitrogen harvest index (NHI) (**b**) under different treatments. C: Controlled irrigation, F: flooded irrigation. Different lowercase letters indicate significant differences among treatments within the same year (*p* < 0.05). Under each irrigation regime, four nitrogen application rates of 0 (N0), 82.5 (N1), 110 (N2), and 137.5 (N3) kg N·ha^−1^ were applied.

Under C, the HI was consistently higher than that under F, with the difference being more pronounced under the N2 treatment (Table 7). As the nitrogen application rate increased, the HI initially increased and then declined, reaching the highest value at the moderate nitrogen application rate (N2) (Figure 3).

Compared with the HI, the NHI showed a more sensitive response to treatment differences. At the same nitrogen application rate, the NHI under C was significantly higher than that under F, indicating enhanced nitrogen allocation to grain under C. Similar to the HI, the NHI increased with increasing nitrogen application rate but declined under N3 treatment, indicating that excessive nitrogen input reduced the proportion of nitrogen translocated to harvestable organs.

## 3. Discussion

### 3.1. Effects of Nitrogen Application Rate and Irrigation Regime on Rice Production

As the nitrogen application rate increased, the rice aboveground dry matter accumulation and harvest index (HI) initially increased and subsequently declined, indicating the presence of an optimal nitrogen range for rice growth. Excessive nitrogen application did not continuously promote panicle growth [20,21]. Instead, it intensified competition for photosynthates among vegetative organs such as stems and leaves, resulting in greater allocation of carbon and nitrogen assimilates to structural tissues and reduced assimilate supply to the panicle [7,22]. Previous studies have reported that under high nitrogen application rates, delayed leaf senescence and enhanced metabolic activity in vegetative organs reduce the efficiency of carbon and nitrogen remobilization to grains during the late growth stages. Consequently, dry matter tends to be retained in stems and leaves rather than translocated to the panicle [14]. This mechanism helps explain the increase–decrease pattern of panicle dry matter accumulation and rice yield observed with increasing nitrogen application in the study. Therefore, selecting appropriate nitrogen application rates is crucial to achieving long-term stability in rice growth while optimizing resource use efficiency.

Rice yield and harvest index were also significantly influenced by the irrigation regime [23]. At the same nitrogen application rate, the HI under controlled irrigation was higher than that under flooded irrigation, indicating that water-saving irrigation favored greater allocation of dry matter to grain. Previous studies have shown that increasing irrigation amounts can reduce the remobilization and translocation efficiency of stored assimilates in rice stems and sheaths from seedling to maturity, leading to a lower HI [24,25]. In contrast, water-saving irrigation enhances the translocation of non-structural carbohydrates, which may be attributed to improved photosynthate utilization efficiency and promoted panicle growth, thereby increasing the HI [26]. The irrigation regime also played a key role in regulating rice yield responses to nitrogen application [27]. Under flooded irrigation, rice yield increased with increasing nitrogen input but tended to plateau at the high nitrogen application rate (N3). Under controlled irrigation, however, yield increased initially and then declined as nitrogen application increased, with yield reductions observed in several years under high nitrogen application rates. This pattern indicates a greater sensitivity of rice yield to nitrogen application rate under controlled irrigation. A possible explanation is that under controlled irrigation, frequent fluctuations in rhizosphere redox conditions may enhance root nitrogen uptake and assimilation at moderate nitrogen application rates but could impose a greater metabolic burden on roots under excessive nitrogen supply [28,29]. This, in turn, could further limit the efficient conversion of photosynthates into reproductive growth [10,30]. By comparison, the relatively stable anaerobic conditions under continuous flooding may partially buffer the effects of high nitrogen application rate on rhizosphere nitrogen transformation, resulting in yield stagnation rather than a clear decline at high nitrogen application rate [31].

Although flooded irrigation achieved higher peak yields in some years under high nitrogen application rate, controlled irrigation combined with moderate nitrogen application was generally associated with a higher HI and a dry matter partitioning pattern favoring grain yield across multiple years [15,17]. Improving rice production efficiency requires optimizing the combination of irrigation regime and nitrogen application [32]. Controlled irrigation combined with moderate nitrogen input enhances the remobilization of dry matter and nitrogen to the panicle and is more effective for achieving stable and efficient rice production than relying on high nitrogen input alone.

### 3.2. Responses of Nitrogen Accumulation and Inter-Organ Partitioning to Nitrogen Application Rate and Irrigation Regimes

Nitrogen application rate exerted a significant influence on nitrogen accumulation in rice plants. Under the same irrigation regime, nitrogen accumulation under the N1 and N2 treatments was higher than that under N0. However, under the N3 conditions, nitrogen accumulation no longer increased with increasing nitrogen application rate but instead often reached a plateau or even declined. Overall, among the nitrogen application treatments, N2 showed relatively higher nitrogen accumulation. Previous studies have shown that excessive external nitrogen supply can increase the metabolic burden in the rice rhizosphere, inhibit carbon–nitrogen assimilation, and constrain plant nitrogen uptake capacity [7,22,33]. This is consistent with the limited nitrogen accumulation observed under the high nitrogen treatment (N3) in the study. Moderate nitrogen application rate enhanced plant nitrogen accumulation, whereas further increases in nitrogen input did not lead to a continuous improvement in nitrogen uptake capacity [34].

The irrigation regime also played an important role in regulating plant nitrogen accumulation [35]. At the N2 nitrogen application rate, plant nitrogen accumulation under controlled irrigation was generally higher than that under flooded irrigation [36]. This response is likely related to improved soil aeration under water-saving irrigation. Controlled irrigation may increase oxygen availability in the rhizosphere, thereby potentially enhancing the activity of enzymes involved in nitrogen transport and assimilation, accelerating organic matter mineralization, and temporarily enlarging the pool of plant-available nitrogen, which together could promote root nitrogen uptake and increase plant nitrogen accumulation [10].

Although plant nitrogen accumulation increased under moderate nitrogen application rate and controlled irrigation, the extent to which this nitrogen can be translated into yield advantages depends on its distribution among plant organs [15]. In the study, the panicle acted as the dominant nitrogen sink, accounting for 70% to 85% of total plant nitrogen. As nitrogen application increased from N0 to N2, the proportion of nitrogen allocated to the panicle increased, accompanied by a higher NHI and AE. In contrast, under a high nitrogen application rate (N3), a greater proportion of nitrogen was retained in vegetative organs such as stems and leaves, leading to declines in panicle nitrogen proportion, NHI, and AE. These results indicate diminishing marginal returns of nitrogen fertilizer in rice production, with only moderate nitrogen application favoring efficient nitrogen translocation from vegetative to reproductive organs [37]. This finding agrees with previous reports showing that efficient remobilization of stored nitrogen from vegetative organs is critical for grain protein formation and yield improvement [15].

Moderate increases in nitrogen input can accelerate nitrogen translocation from vegetative tissues, whereas stable high yields depend on effective nitrogen remobilization during the grain-filling period [38]. Excessive nitrogen applications not only reduce physiological nitrogen use efficiency but may also suppress grain formation [39]. This is likely because excessive nitrogen stimulates vigorous vegetative growth and delays leaf senescence, diverting assimilates toward protein turnover and structural tissue formation and reducing nitrogen transport to grains [14].

Compared with flooded irrigation, controlled irrigation resulted in a higher NHI and AE, particularly under the N1 and N2 treatments. This indicates that controlled irrigation favors nitrogen allocation to grains rather than retention in vegetative organs, likely through improved coordination of carbon and nitrogen metabolism. Controlled irrigation improves soil aeration conditions [40]. This increased aeration contributes to creating a more oxidative environment around the roots. Enhanced soil aeration in paddy fields leads to an improved oxidative status of rice roots and may enhance the activity of key nitrogen-metabolizing enzymes such as nitrate reductase (NR) and glutamine synthetase (GS) [41], thereby promoting organic nitrogen mineralization and inorganic nitrogen uptake and maintaining root nitrogen absorption and assimilation at levels favorable for grain [28]. Previous studies have also shown that water-saving irrigation induces more dynamic soil redox conditions, enhances root nitrogen uptake, improves nitrogen remobilization efficiency, and promotes the translocation of carbon and nitrogen compounds to grains, particularly during the grain-filling stage. In contrast, long-term flooded irrigation tends to stimulate vegetative growth while constraining reproductive development, increasing nitrogen retention in stems and leaves, and ultimately reducing nitrogen harvest index [42,43].

Overall, controlled irrigation maintains rice yield not by simply increasing total nitrogen uptake, but by improving nitrogen partitioning efficiency among plant organs, especially by enhancing nitrogen remobilization to the panicle. Under moderate nitrogen input, this mechanism contributes to relatively high rice productivity across most study years, as reflected by the interannual responses of NHI and AE.

Despite the robust multi-year field evidence provided in this study, several limitations should be acknowledged. Soil nitrogen transformation processes and root physiological responses under contrasting irrigation regimes were not directly measured, and therefore, the proposed mechanisms regarding rhizosphere redox regulation and nitrogen assimilation are inferred from established literature rather than directly verified in this experiment. Furthermore, the experiment was conducted at a single site with specific soil and climatic conditions, and the general applicability of the findings to other rice-growing regions with different soil types or water management systems requires further validation.

Future research should directly quantify nitrogen remobilization dynamics under controlled irrigation. Additionally, coupling soil nitrogen transformation measurements with root physiological indicators would help clarify the mechanistic pathways linking irrigation regimes to nitrogen partitioning efficiency. Multi-location experiments across diverse agroecological zones are also necessary to evaluate the scalability of the proposed water–nitrogen optimization strategy. Such efforts would further strengthen the mechanistic understanding and practical applicability of irrigation–nitrogen management for sustainable rice production.

## 4. Materials and Methods

### 4.1. Site Description

The field experiment was conducted at the Qing’an National Irrigation Experimental Station, located in Heilongjiang Province, northeastern China (127°40′45″ E, 46°57′28″ N). The region is characterized by a cold temperate continental monsoon climate, with a mean annual precipitation of approximately 550 mm and a mean annual surface evaporation of about 750 mm. The mean annual air temperature is 2.5 °C, and the annual solar radiation ranges from 4000 to 4300 MJ m^−2^. The site is located in the transition zone between the second and third thermal zones of China, with an annual accumulated temperature of approximately 2532 °C·d. The rice growing season generally lasts 110–130 days, and the frost-free period is approximately 128 days. The soil is classified as Mollisols according to the U.S. Department of Agriculture (USDA) soil taxonomy. The soil texture of this region is classified as sandy clay, and the physical-chemical properties of the 0–20 cm layer are shown in Table 8.

### 4.2. Experimental Design

The long-term field experiment was established in 2017 and has been continuously maintained since then. Yield and plant sampling were conducted in 2021–2024. The experiments were conducted using the rice variety “Suijing 18” consistently across all study years. The experiment followed a randomized complete block design, consisting of eight treatments with three replicates (Table 9). Two irrigation regimes were implemented: controlled irrigation (C) and flooded irrigation (F). Under each irrigation regime, four nitrogen application rates of 0 kg N·ha^−1^ (N0), 82.5 kg N·ha^−1^ (N1), 110 kg N·ha^−1^ (N2), and 137.5 kg N·ha^−1^ (N3) were applied, corresponding to 0%, 75%, 100%, and 125% of the locally recommended nitrogen application rates respectively. The selected nitrogen rates were determined according to local agronomic guidelines and validated by previous long-term field studies in the study area.

Each plot covered an area of 100 m^2^ (10 m × 10 m). Rice seedlings were transplanted at 30 cm × 10 cm spacing with three seedlings per hill. To minimize lateral movement of water and nutrients among plots, plastic film barriers were installed vertically along all plot boundaries to a depth of 40 cm.

Nitrogen fertilizer was applied as urea (The nitrogen content of the urea fertilizer used in this study is 46.6%) in three split applications. Nitrogen fertilizer was split-applied at the ratios of 45%, 20%, and 35% of the total amount as basal fertilizer, tillering fertilizer, and panicle fertilizer, respectively. The basal N fertilizer was applied one day before transplanting, the tillering fertilizer at 14 days after transplanting, and the panicle fertilizer at 45 days after transplanting. Phosphorus was applied at 45 kg P·ha^−1^ as a basal fertilizer before transplanting. Potassium was applied at 80 kg K·ha^−1^, with 50% applied as a basal fertilizer and the remaining 50% at the 8.5 leaf stage [45]. Weed, pest, and disease management followed local agronomic recommendations.

Irrigation treatments were implemented throughout the rice-growing season. Under flooded irrigation, a standing water layer of 30–50 mm was maintained during most growth stages, with mid-season drainage at the late tillering stage and natural drainage at maturity. Under controlled irrigation, a shallow water layer (0–30 mm) was maintained only during regreening and early tillering stages. Thereafter, the field surface was kept non-flooded, and irrigation was scheduled based on soil water content in the root zone. Soil water content and surface water depth were monitored twice daily using a TRIME-PICO64/32 time-domain reflectometry meter (IMKO, Ettlingen, Germany) and a field ruler, respectively. Soil volumetric water content in the root zone was monitored using a sensor installed at a depth of 15 cm. The sensor was calibrated against gravimetrically determined soil moisture content using field soil samples prior to installation. These threshold values were selected based on preliminary field trials and previous studies conducted at the same experimental site. Irrigation was applied when soil water content reached the lower threshold and subsequently restored to the upper threshold specified for each growth stage (Table 10) [45].

### 4.3. Sampling and Measurements

At the rice maturity stage during 2021–2024, rice plants were randomly collected from the inner area of each plot. Each treatment was set up with three replicates. Samples were taken from five sampling points within each plot and combined to form one composite sample per replicate. A 0.15 m^2^ area was selected within a uniform section of the plot, and plants were cut at ground level. Samples were separated into stems, leaves, and panicles, oven-dried at 105 °C for 30 min, followed by drying at 80 °C to constant weight, and the dry weight was determined. Aboveground dry matter accumulation was calculated as the sum of dry matter of stems, leaves, and panicles. The dried samples were then ground and passed through a 1 mm sieve for the determination of N concentrations using the Kjeldahl [46].

Grain yield was determined at maturity using a five-point sampling method. In each plot, plants were harvested from five randomly selected areas (each area 60 cm × 60 cm). After threshing, grains were air-dried, and the yield was adjusted to a standard moisture content of 14% to ensure comparability among treatments. After threshing, grains were air-dried and weighed to obtain grain yield.

### 4.4. Parameter Calculation

#### 4.4.1. Nitrogen Accumulation and Distribution

Nitrogen accumulation in each organ (*Nacc*, *organ*, kg N·ha^−1^) was calculated as Equation (1):

(1) Nacc,organ=Nc×DW

where *Nc* is the nitrogen concentration in each organ (%), the value of *Nc* ranges between 0 and 1, and *DW* is the dry weight of the organ corresponding (kg·ha^−1^).

Total aboveground N accumulation (*Nacc*, *total*, kg N·ha^−1^) was calculated as Equation (2) [47]:

(2) Nacc,total=Σ Nacc,organ

The *N distribution ratio* in each organ (%) was calculated as Equation (3):

(3) $$N\;distribution\;ratio=\frac{Nacc,organ}{Nacc,total}\;\times \;100\%$$

The AE reflects the increase in grain yield per unit of nitrogen fertilizer applied and is a key indicator for assessing the effectiveness of nitrogen input. The agronomic efficiency of nitrogen fertilizer (*AE*, kg·kg^−1^ N) was calculated according to Equation (4) [48]:

(4) $$AE=\frac{\left(Yf-Y0\right)}{Nappl}$$

where *Yf* is grain yield under the different treatments (kg·ha^−1^), *Y*0 is grain yield in the unfertilized control (kg·ha^−1^), and *Nappl* is the applied nitrogen rate (kg N·ha^−1^).

#### 4.4.2. Calculation of Harvest Index and Nitrogen Harvest Index

The HI, which reflects the efficiency of converting photosynthates into economic yield, and the NHI, which represents the proportion of total plant nitrogen allocated to the harvested parts.

The harvest index (*HI*) and nitrogen harvest index (*NHI*) were calculated as Equations (5) and (6):

(5) $$HI\left(\%\right)=\frac{GY}{DM}\times 100$$

(6) $$NHI\left(\%\right)=\frac{Ngrain}{Ntotal}\times 100\%$$

where *GY* is grain yield (kg·ha^−1^), and *DM* is the total aboveground dry matter accumulation. *Ngrain* is nitrogen accumulation in grain (kg N·ha^−1^), and *Ntotal* is the total nitrogen accumulation in the aboveground part of the plant (kg N·ha^−1^) [49].

### 4.5. Statistical Analysis

Microsoft Excel 2019 (Microsoft Corp., Redmond, WA, USA) was used to conduct preliminary statistical processing of experimental data. Origin 2021 (Origin Lab Corp., Northampton, MA, USA) was used for plotting. Normality of residuals was assessed using the Shapiro–Wilk test, and homogeneity of variances was evaluated using Levene’s test. When necessary, data were log- or square-root transformed prior to analysis; however, untransformed means are presented for clarity. A three-way analysis of variance (ANOVA) was performed to evaluate the main effects of irrigation regime (I), N application rate (N), and year (Y), as well as their interaction effects (I × N, I × Y, N × Y, and I × N × Y) on yield, dry matter accumulation, nitrogen accumulation. One-way ANOVA was conducted to assess the differences among treatments, and the significance of differences among the treatments was analyzed using Tukey’s post hoc test method, with *p* < 0.05. All statistical analyses were conducted using IBM SPSS Statistics version 27 (IBM Corp., Armonk, NY, USA).

## 5. Conclusions

This study demonstrated that increasing N application enhanced rice yield and N accumulation up to a moderate level, beyond which yield plateaued or declined, and agronomic N use efficiency decreased. Although flooded irrigation occasionally produced slightly higher yields under high N input, controlled irrigation combined with 110 kg N ha^−1^ resulted in a higher harvest index and nitrogen harvest index, reflecting improved nitrogen allocation to the panicle. These findings suggest that optimizing nitrogen partitioning under controlled irrigation can improve agronomic N use efficiency without increasing N input beyond moderate levels. Overall, controlled irrigation combined with 110 kg N ha^−1^ provided a favorable balance between yield performance and nitrogen use efficiency under the conditions of this study.

## Figures and Tables

**Table 1:** Analysis of variance of rice yield under different water and N management practices.

Source of Variation	I	N	Y	I × N	I × Y	Y × N	I × N × Y
ANOVA	*	**	ns	**	ns	*	**

Note: I, irrigation regime; N, nitrogen application rate; Y, year. I × N, I × Y, N × Y, and I × N × Y represent the corresponding interaction effects. Significance levels are indicated as follows: ns, not significant; * *p* < 0.05; ** *p* < 0.01. Statistical analysis was performed using factorial ANOVA.

**Table 2:** Dry matter accumulation in the aboveground parts of rice under different treatments.

Years	Treatments	Stem (kg·ha^−1^)	Leaf (kg·ha^−1^)	Panicle (kg·ha^−1^)	Rice Aboveground Dry Matter Accumulation (kg·ha^−1^)
2021	FN0	2938.51 ± 24.68 d	731.40 ± 32.94 d	5216.14 ± 120.00 d	8886.05 ± 126.84 c
	FN1	3757.78 ± 133.26 ab	873.96 ± 68.78 ab	5395.95 ± 210.50 d	10,027.69 ± 408.95 b
	FN2	3731.62 ± 136.05 a	927.28 ± 23.61 a	5694.08 ± 35.81 d	10,352.98 ± 136.86 a
	FN3	3309.64 ± 54.14 bc	806.80 ± 19.60 bc	6021.34 ± 98.68 c	10,137.78 ± 34.11 b
	CN0	2897.72 ± 44.25 d	706.53 ± 17.08 d	5372.94 ± 143.24 d	8977.18 ± 170.34 c
	CN1	3159.24 ± 82.20 c	748.13 ± 7.04 cd	6822.67 ± 138.09 a	10,730.23 ± 124.38 a
	CN2	2953.91 ± 19.61 d	715.42 ± 45.07 d	6655.04 ± 151.69 a	10,324.37 ± 196.37 a
	CN3	3441.44 ± 175.84 b	819.57 ± 11.29 b	6002.45 ± 96.28 b	10,263.46 ± 251.99 ab
2022	FN0	4011.73 ± 48.48 d	1153.87 ± 24.88 b	7500 ± 209.27 d	12,665.60 ± 163.67 b
	FN1	4508.80 ± 101.02 a	1144.00 ± 120.95 b	7781.07 ± 315.29 d	13,433.87 ± 323.00 b
	FN2	4396.20 ± 21.58 b	1109.60 ± 23.62 be	9672.00 ± 100.00 a	15,177.60 ± 96.29 a
	FN3	4005.60 ± 7.06 d	1043.73 ± 15.96 cd	9633.33 ± 159.08 a	14,682.66 ± 179.97 a
	CN0	4488.80 ± 144.03 b	1425.07 ± 18.95 a	7192.80 ± 135.89 e	13,106.67 ± 291.56 b
	CN1	4415.07 ± 105.24 b	1403.47 ± 18.08 a	7922.13 ± 430.32 d	13,740.67 ± 546.04 ab
	CN2	3946.40 ± 229.99 d	1167.73 ± 18.62 b	9677.33 ± 239.66 b	14,791.46 ± 105.07 a
	CN3	3804.18 ± 27.03 d	992.27 ± 16.66 d	8542.40 ± 147.50 d	13,339.47 ± 189.13 b
2023	FN0	4635.47 ± 168.65 d	1206.67 ± 14.73 a	6477.60 ± 197.39 f	12,319.74 ± 51.28 d
	FN1	4303.73 ± 219.25 f	1192.53 ± 9.81 a	8955.73 ± 112.61 b	14,451.99 ± 248.28 bc
	FN2	4942.93 ± 85.73 cd	1009.33 ± 40.71 b	8945.60 ± 198.63 b	14,897.86 ± 114.41 b
	FN3	4827.73 ± 46.56 d	976.53 ± 17.64 bc	8577.60 ± 161.48 bc	14,381.86 ± 214.57 bc
	CN0	5156.27 ± 166.77 bc	872.21 ± 78.51 c	7164.8 ± 530.74 e	13,192.05 ± 126.84 c
	CN1	5353.33 ± 159.08 ab	930.13 ± 78.63 bc	8062.13 ± 168.59 cd	14,345.69 ± 408.95 bc
	CN2	5518.93 ± 117.68 a	1162.93 ± 15.13 a	9156.8 ± 242.71 a	15,836.78 ± 34.11 a
	CN3	5021.87 ± 122.77 cd	949.33 ± 15.79 bc	7509.6 ± 286.67 de	13,479.98 ± 136.86 c
2024	FN0	3624.01 ± 313.07 bc	1013.60 ± 24.81 cd	8024.48 ± 228.93 ab	12,661.19 ± 170.34 c
	FN1	3624.21 ± 249.31 bc	1132.80 ± 17.96 a	8828.88 ± 558.98 a	13,584.04 ± 124.38 b
	FN2	5064.14 ± 915.15 ab	1062.40 ± 14.97 b	8724.82 ± 174.29 a	14,850.37 ± 196.37 a
	FN3	3272.14 ± 196.29 c	939.20 ± 20.58 e	9149.46 ± 471.20 a	13,360.46 ± 251.99 b
	CN0	4960.51 ± 803.51 ab	1050.40 ± 28.42 bc	6860.87 ± 893.63 e	12,870.6 ± 163.67 bc
	CN1	5520.31 ± 496.90 a	1065.60 ± 22.26 b	7717.63 ± 312.84 ab	13,433.87 ± 323.00 b
	CN2	5616.47 ± 937.54 a	982.40 ± 13.05 d	8024.85 ± 803.58 ab	14,622.66 ± 179.90 a
	CN3	4584.14 ± 778.68 b	996.58 ± 14.71 d	8802.47 ± 725.65 a	14,382.6 ± 96.29 a

Note: Each value represents the average ± standard error (n = 3); the different lowercase letters indicate statistically significant differences between treatments (*p* < 0.05). C: Controlled irrigation, F: flooded irrigation. Under each irrigation regime, four nitrogen application rates of 0 (N0), 82.5 (N1), 110 (N2), and 137.5 (N3) kg N·ha^−1^ were applied.

**Table 3:** Analysis of variance of components of rice dry matter under different water and N management practices.

Source of Variation	Stem	Leaf	Panicle	Rice Aboveground Dry Matter Accumulation
I	*	ns	*	*
N	*	**	*	**
Y	**	**	**	**
I × N	**	ns	*	**
I × Y	ns	*	**	*
Y × N	*	**	*	*
I × N × Y	**	*	**	**

Note: I, irrigation regime; N, nitrogen application rate; Y, year. I × N, I × Y, N × Y, and I × N × Y represent the corresponding interaction effects. Significance levels are indicated as follows: ns, not significant; * *p* < 0.05; ** *p* < 0.01. Statistical analysis was performed using factorial ANOVA.

**Table 4:** Total rice aboveground nitrogen accumulation under different treatments.

Aboveground Nitrogen Accumulation (kg·ha^−1^)				
Treatments	2021	2022	2023	2024
FN0	63.76 ± 2.05 c	66.22 ± 1.95 d	86.45 ± 0.82 cd	79.95 ± 1.12 e
FN1	67.84 ± 0.65 c	75.37 ± 0.87 c	110.18 ± 2.52 b	111.38 ± 1.20 ab
FN2	92.15 ± 1.09 a	85.45 ± 1.24 bc	108.56 ± 0.95 b	98.45 ± 0.89 c
FN3	81.52 ± 2.47 b	88.32 ± 1.14 bc	91.63 ± 1.09 c	104.29 ± 1.10 c
CN0	78.64 ± 0.99 b	75.84 ± 1.03 c	90.58 ± 1.00 c	74.20 ± 1.17 e
CN1	77.14 ± 1.04 b	89.69 ± 0.94 b	129.71 ± 1.17 a	89.08 ± 0.59 d
CN2	94.07 ± 1.02 a	109.93 ± 1.06 a	103.95 ± 1.32 b	123.22 ± 7.18 a
CN3	75.86 ± 1.37 b	83.31 ± 1.12 bc	71.83 ± 1.41 d	111.85 ± 1.03 ab

Note: Each value represents the average ± standard error (n = 3); the different lowercase letters indicate statistically significant differences between treatments (*p* < 0.05). C: Controlled irrigation, F: flooded irrigation. Under each irrigation regime, four nitrogen application rates of 0 (N0), 82.5 (N1), 110 (N2), and 137.5 (N3) kg N·ha^−1^ were applied.

**Table 5:** Analysis of variance of components of aboveground nitrogen accumulation under different water and N management practices.

Source of Variation	I	N	Y	I × N	I × Y	Y × N	I × N × Y
ANOVA	**	**	*	**	*	*	**

Note: I, irrigation regime; N, nitrogen application rate; Y, year. I × N, I × Y, N × Y, and I × N × Y represent the corresponding interaction effects. Significance levels are indicated as follows: ns, not significant; * *p* < 0.05; ** *p* < 0.01. Statistical analysis was performed using factorial ANOVA.

**Table 6:** The agronomic efficiency of nitrogen fertilizer under different treatments.

The Agronomic Efficiency of Nitrogen Fertilizer (kg·kg^−1^ N)				
Treatments	2021	2022	2023	2024
FN0	/	/	/	/
FN1	1.85 ± 0.45 d	3.15 ± 0.31 c	32.79 ± 8.34 a	10.43 ± 2.31 ab
FN2	4.53 ± 0.36 c	22.32 ± 2.12 ab	25.88 ± 2.36 a	7.18 ± 0.36 c
FN3	7.71 ± 0.61 c	17.61 ± 2.36 b	17.34 ± 2.34 b	9.42 ± 1.31 b
CN0	/	/	/	/
CN1	19.33 ± 2.36 a	12.59 ± 1.36 b	11.64 ± 5.36 c	11.74 ± 1.39 ab
CN2	13.70 ± 1.30 b	27.44 ± 3.65 a	16.35 ± 1.02 b	17.40 ± 0.64 a
CN3	5.16 ± 0.50 c	12.16 ± 0.64 b	3.92 ± 0.96 d	9.77 ± 0.34 b

Note: Each value represents the average ± standard error (n = 3); the different lowercase letters indicate statistically significant differences between treatments (*p* < 0.05). C: Controlled irrigation, F: flooded irrigation. Under each irrigation regime, four nitrogen application rates of 0 (N0), 82.5 (N1), 110 (N2), and 137.5 (N3) kg N·ha^−1^ were applied.

**Table 7:** Analysis of variance of components of HI and NHI under different water and N management practices.

Source of Variation	I	N	Y	I × N	I × Y	Y × N	I × N × Y
HI ANOVA	*	**	ns	**	ns	*	**
NHI ANOVA	**	**	*	**	**	**	**

Note: I, irrigation regime; N, nitrogen application rate; Y, year. I × N, I × Y, N × Y, and I × N × Y represent the corresponding interaction effects. Significance levels are indicated as follows: ns, not significant; * *p* < 0.05; ** *p* < 0.01. Statistical analysis was performed using factorial ANOVA.

**Table 8:** Physico-chemical attributes of the soil in the experimental area.

Soil Properties	
pH	5.81
Field capacity (%)	31.5
Total porosity (%)	55.6
Organic matter (g·kg^−1^)	32.7
Total N (g·kg^−1^)	1.4
Available P (mg·kg^−1^)	36.2
Available K (mg·kg^−1^)	112.1
Cation exchange capacity (cmol·kg^−1^)	31.4
Soil texture	Sandy clay loam

Note: Analytical methods for soil properties were performed according to the procedures outlined in Carter & Gregorich (2008), Soil Sampling and Methods of Analysis (Canadian Society of Soil Science) [44].

**Table 9:** Experimental treatments.

Treatments	Irrigation Regimes	Nitrogen Application Rates (kg N·ha^−1^)
FN0	Flooded irrigation	0.0
FN1	Flooded irrigation	82.5
FN2	Flooded irrigation	110.0
FN3	Flooded irrigation	137.5
CN0	Controlled irrigation	0.0
CN1	Controlled irrigation	82.5
CN2	Controlled irrigation	110.0
CN3	Controlled irrigation	137.5

**Table 10:** Different water management patterns at rice growth stages.

Irrigation Regimes	Re-Greening	Early Tillering	Tillering	Later Tillering	Jointing-Booting	Heading-Flowering	Milk	Mature
Controlled irrigation	0–30 mm	0.7 *θ_s_*–*θ_s_*	0.7 *θ_s_*–*θ_s_*	Drainage	0.8 *θ_s_*–*θ_s_*	0.8 *θ_s_*–*θ_s_*	0.7 *θ_s_*–*θ_s_*	Naturally drying
Flooded irrigation	0–30 mm	0–50 mm	0–50 mm	Drainage	0–50 mm	0–50 mm	0–30 mm	Naturally drying

Note: *θ_s_* is the soil saturated water content mass fraction in the root layer. The data before “–” is the lower limit of moisture control, and the data after “–” is the upper limit of moisture control. Depth of field water layer unit: mm.

## Data Availability

The original contributions presented in this study are included in the article. Further inquiries can be directed to the corresponding authors.
